# Oxidative Stability of Protease Treated Peanut with Reduced Allergenicity

**DOI:** 10.3390/foods9060762

**Published:** 2020-06-10

**Authors:** Jianmei Yu, Ivy N. Smith, Nadia Idris, Nicole Gregory, Nona Mikiashvili

**Affiliations:** Department of Family and Consumer Sciences, North Carolina Agricultural and Technical State University, 1601 East Market Street, Greensboro, NC 27411, USA; ivyn.smith@gmail.com (I.N.S.); nyidris@aggies.ncat.edu (N.I.); negregor@aggies.ncat.edu (N.G.); nmikiash@ncat.edu (N.M.)

**Keywords:** peanuts, protease treatment, allergenicity, oxidation, antioxidant activity

## Abstract

Oxidative stability and allergenicity are two major concerns of peanuts. This study evaluated the impact of protease treatment of peanuts on its oxidative stability during storage. The raw and dry-roasted peanut kernels were hydrolyzed with Alcalase solution at pH 7.5 for 3 h. The contents of Ara h 1, Ara h 2, and Ara h 6 in peanuts were determined before and after enzyme treatment by a sandwich ELISA. After drying, the samples were packed in eight amber glass jars and stored at 37 °C for 1–8 weeks. Controls are untreated raw and dry-roasted peanuts packed and stored in the same way as their treated counterparts. Samples were taken biweekly to determine peroxide value (PV) and thiobarbituric acid reactive substances (TBARS) as indicators of oxidation (*n* = 3), and to determine antioxidant activity. Alcalase treatment reduced intact major allergens Ara h 1, Ara h 2, and Ara h 6 by 100%, 99.8%, and 85%, respectively. The PVs of Alcalase-treated raw and roasted peanuts was lower than those of untreated (*p <* 0.05) over the 8-week storage. The TBARS of Alcalase-treated raw peanuts were slightly higher than that of untreated (*p <* 0.05), but the TBARS of Alcalase-treated dry-roasted peanuts were slightly but significantly lower than that of untreated (*p <* 0.05). The protease treatment increased the antioxidant activities including reducing power, DPPH free radical scavenging capacity, and metal chelating capacity of peanuts.

## 1. Introduction

Peanut is a nutrient balanced food product. It contains 20.7–25.3% of protein, 50–55% of crude fat, 15.8–20.9% carbohydrates including 8.5–9.5% fiber; it is also rich in vitamins and minerals [1]. Regular peanut consumption has been reported to be adversely associated with the risks of cancer, cardiovascular, respiratory, infectious, renal, and liver disease mortality but not with diabetes or Alzheimer’s disease mortality [2,3,4,5]. Several cross-sectional analyses and some independent prospective studies have shown an inverse association between long-term frequent nut-eating habit and lower body weight [6,7,8]. The B vitamins, tocopherols, mono-/poly-unsaturated fatty acids, dietary fiber, and phytochemicals in peanuts are considered the compounds responsible for the protective effects [9].

Due to the high content (up to 50%) of lipids, the oxidative stability during storage is a major concern of peanuts, particularly, after roasting because of the high unsaturated fat content. Normal-oleate peanut genotypes consist 36–69% oleic acid and 13–40% linoleic acid, whereas high-oleate peanut genotypes have 80% oleic acid, 2% linoleic acid, and the total unsaturated fatty acids of peanut oil is about 82% [10,11]. In the presence of oxygen, unsaturated fatty acids are oxidized to form odorless, tasteless hydroperoxides. The quantification of hydroperoxide provides an estimate of the overall oxidation status for lipids and lipid-containing foods in the primary phase or induction period of oxidation [12]. The traditional method for quantification of hydroperoxides is the determination of peroxide value (PV) [13]. The hydroperoxides are relatively unstable and they decompose to form different aldehydes such as hexanal, 4-hydroxynonenal (HNE), and malondialdehyde (MDA), formaldehydes, and acetaldehydes which are perceived as off-flavors and as a warning that food is no longer edible [14]. All types of aldehydes react with TBA to form colored compounds.

Generally, peanuts are roasted to enhance their flavor, to destroy the antinutritional factors, and to reduce or eliminate the spoilage and pathogenic microorganisms of raw peanuts [15,16]. Roasted peanuts were oxidized faster than raw peanuts, and peanuts roasted properly for human consumption were oxidized more rapidly during storage than those roasted insufficiently or excessively [17,18]. Oxidation of lipid in peanuts, particularly roasted peanuts, limits the shelf life, reduces the nutritional value, and renders peanuts unacceptable by consumers due to the rapid development of rancidity [18,19,20]. Research has shown that the oxidation speed of peanuts is significantly affected by oxygen concentration, moisture, and packaging materials and packaging methods [17,21]. Different means have been reported to extend the shelf life of roasted peanuts to various degrees. As the main mechanism of peanut oxidation is free radical chain reaction, reducing oxygen concentration by modified atmosphere packing such as nitrogen filling, vacuum packaging, UV-proof packaging, and blocking oxygen penetration by edible coating [22,23] were reported, effectively slowing down the rancidity development caused by oxidation.

Although consumption of peanuts has been associated with many health benefits, it also raises a serious food safety issue for individuals with peanut allergy. About 2.5% children and 1.8% of adults in the U.S. are allergic to peanut [24,25]. The prevalence of peanut allergy in Europe is 2.2% [26]. The allergic reaction is triggered by allergenic proteins which account for 85% of total proteins in the peanuts [27]. Agricultural and food scientists have made significant effort to reduce the allergenic potential of peanuts. Some previous studies demonstrated that protease hydrolysis of peanuts or peanut flour is an effective post-harvest approach to mitigate the allergenicity of peanuts [28,29,30,31,32]. However, the breaking down of protein molecules by proteolytic enzyme modifies the surface and internal structure of peanut kernels, which may result in leaching of antioxidant vitamin E originally present in peanut, thus changing in oxidative stability. On another side, limited studies showed that the breakdown of peanut protein by protease produced antioxidant peptides [33,34].

Based on the above description, there is a need to evaluate the oxidation stability of protease treated peanut kernels. Our previous study found that Alcalase was more effective than other proteases in reducing the allergen content and overall allergenicity of peanuts [30]. Therefore, this study evaluated the impact of Alcalase treatments on the oxidative stability of raw peanuts and dry-roasted peanuts during an 8-week storage period at 37 °C using peroxide value (PV), and thiobarbituric acid reactive substances (TBARS) and as indicators of oxidation. The study also compared the antioxidant activities of protein extracts from Alcalase treated and untreated peanut kernels.

## 2. Materials and Methods

Raw and dry roasted Runner peanuts were purchased online from The Peanut Patch. Alcalase (2.4 L, Novozyme, Bagsvaerd, Denmark), sodium hydroxide, sodium thiosulfate, potato starch, potassium iodine, trichloroacetic acid (TCA), hexane, methanol, 2, 2-diphenyl-1-picrylhydrazyl (DPPH•), BCA reagents, iron dichloride (FeCl_2_), potassium ferricyanide, ferrozine were purchased from Fisher Scientific (Waltham, MA, USA). Ferric Chloride (FeCl_3_) and thiobarbituric acid (TBA) were purchased from Sigma-Aldrich (St. Louis, MO, USA)

### 2.1. Enzymatic Treatment

Raw and dry roasted peanut kernels (600 g each) were treated with 3.5% Alcalase (3.5 mL of Alcalase per 100 g peanuts) in phosphate buffer (pH 7.5, 20 mM) for 3 h at 40 °C, then vacuum dried at 75 °C for 18 h. After cooling at room temperature, a small quantity of each peanut sample was used for allergen, allergenicity, and antioxidant activity tests, the rest was used for oxidative stability experiments. Untreated raw and roasted peanuts were used as controls.

### 2.2. Protein Extraction and Quantification

The soluble protein was extracted and quantified as previously described [32]. Briefly, peanut samples were ground into butters and stored at −20 °C. The soluble protein of peanut butter sample was extracted using phosphate buffer (pH 8.0, 20 mM). The extraction of each sample was conducted twice at peanut to buffer ratio of 1:20 (*w*/*v*) at ambient temperature under constant stirring, each 60 min, followed by centrifugation at 3000 g for 20 min using 5810R centrifuge (Eppendorf North America, Hauppauge, NY, USA). The combined supernatant was centrifuged again and the fat layer on the top was removed. The protein concentrations of extracts were determined by Bicinchoninic acid (BCA) method using a Pierce BCA Protein Assay Kit (Rockford, IL, USA) as described previously and expressed as mg protein per gram peanuts.

### 2.3. Determination of Major Allergen Contents of Protease Treated Peanuts

The concentrations of major allergens Ara h 1, Ara h 2, and Ara h 6 in peanut extracts were determined using sandwich ELISA kits (Indoor Biotechnologies, Charlottesville, VA, USA) according to manufacturer’s instructions as previously described [32]. Each kit includes allergen specific primary antibody, secondary antibody, and allergen standard solution. Other reagents needed for the assays but not provided in the kits were Streptavidin-Peroxidase, and Peroxidase-conjugated Goat Anti-Rabbit and 1 mM ABTS and they were purchased from Sigma-Aldrich (St. Louis, MO, USA). The extracts were diluted to various degrees with phosphate buffer saline (PBS) to ensure the allergen concentration within the detection range of the kit. Each allergen analysis was conducted in triplicate for the same sample. Results were expressed as μg allergen per gram peanuts according to dilution factor, the volume of extract, and sample weight.

### 2.4. Evaluation of In Vitro Allergenicity of Peanuts

The ability of a peanut protein to bind the IgE-antibody in the plasma of peanut allergic patient is an indicator of its allergenic potential. The IgE-binding of peanuts extracts and their corresponding precipitates were tested by Western blot as described in our previous study [30]. Briefly, the protein extracts were diluted with PBS to 1 mg/mL the proteins which were loaded to 12%/4% acrylamide gel (25 mg/mL), resolved using a MiniProtein 3 Cell electrophoresis unit (Bio Rad, Hercules, CA, USA), and then transferred from the unstained SDS-PAGE gel to nitrocellulose membrane using Trans-Blot^®^ Turbo Blotting System (Bio-Rad, Hercules, CA, USA). After blocking with 3% BSA-TBST (Tween 20 in tris buffer saline) for 1 h, the membrane was incubated with the pooled plasma (ImmunoCAP > 100, 1:60 diluted before use) of six peanut allergic patients (PlasmaLab International, Everett, WA, UAS) for 1 h with gentle agitation at ambient temperature. The membrane was then washed and incubated with properly diluted goat anti-human IgE peroxidase-conjugated antibody for 1 h, and then washed three times with TBST. The blot was developed with ECL substrate (Pierce, Rockford, IL, USA) according to the manufacturer’s instructions. The image of blot was taken using an Amersham Imager 600 (GE Healthcare, South Plainfield, NJ, USA).

### 2.5. Storage Stability Test

A 2 × 2 × 9 factorial design was used for the storage stability study and total 36 experiments were conducted. The three factors are type of peanuts (raw and roasted), treatment (untreated and protease treated), and storage time (0–8 weeks). Enzyme treated and untreated peanuts were packed in glass jars (50 g/jar, nine jars per sample) and capped with lids. The jars were placed in an incubator at 37 °C for 0–8 weeks. Peanut samples were removed weekly from incubator and ground into butter for oxidation evaluation.

### 2.6. Evaluation of Oxidation Status

The oxidation status of protease treated and untreated peanuts were evaluated using their peroxide value (PV) and thiobarbituric acid (TBA) reactive substance (TBARS) as indicators. For PV determination, peanut oil was extracted from 30 g peanut butter using hexane containing 0.2% BHA/BHT. After removing hexane by evaporation using an R-300 Rotovapor (Büchi), the PV of the extracted oil was determined by AOAC method 965.33 [35] and expressed as meq O_2_/kg oil. Briefly, 2.50 g of peanut oil was weighed into 125 mL flask followed by adding 15 mL of acid acetic-chloroform solution, 0.25 mL of saturated potassium iodide (KI) solution, 15 mL of DI water, and 0.5 mL of 1% starch solution. The mixture was mixed immediately, and then titrated with a 0.01 N sodium thiosulfate solution under constant stirring until the endpoint was reached (the blue-purple color disappeared). The amount (mL) of sodium thiosulphate solution consumed was recorded and used to calculate PV. The measurement was conducted in triplicate for each sample.

TBARS was determined by a direct TBA assay. TBARS was determined using peanut butter by the method described by Papastergiadis and colleagues [36] with slight modification and expressed as mg MDA/kg peanut. Briefly, 5.00 g of peanut butter was mixed with 25 mL of ice cooled 7.5% TCA solution containing 0.10% propylgallate and 0.10% EDTA. After homogenization using a PT-MR 2100 Polytron Homogenizer (Kinematica, Bohemia, NY, USA), the mixture was centrifuged at 3000 g for 15 min in a 5810R centrifuge (Eppendorf North America, Hauppauge, NY, USA). For each peanut sample, the extraction was conducted in triplicate. The supernatant was mixed with 4.0 mM TBA reagent (1:3, *v*/*v*) in a test tube. The tube was loosely capped and heated in the boiling water bath for 30 min, cooled with in cold water to room temperature. The reaction mixture was filtered using 0.45 µm glass fiber syringe filter and the absorbance was measured at 530 nm. A standard curve was prepared using malonaldehyde (MDA) solutions in the concentration range of 0.2–10 µM. The results were expressed as mg MDA/kg peanut. The TBARS assay was conducted in triplicate for each peanut sample.

### 2.7. Antioxidant Activity of Protein Extracts of Peanuts

The in vitro antioxidant activities including ferric reducing antioxidant power (FRAP), 2,2-diphenyl-1-picrylhydrazyl free radical (DPPH•) scavenging capacity (FRSC) and metal chelating capacity (MCC) of PPH samples were determined. The FRAP method measures the ability of antioxidants to reduce ferric iron, an oxidation trigger, and the result was expressed by the absorbance at 700 nm. The higher absorbance corresponds to higher reducing power. When the stable DPPH• reacts with a hydrogen donor (antioxidant), the reduced form DPPH is generated accompanied by the disappearance of the violet color, thus the absorbance decreases linearly with the antioxidant concentration [37]. The FRAP and DPPH• scavenging capacity of PPH in the protein concentration range 0.5–2.5 mg/mL, and metal chelating capacity of PPH was determined in the protein concentration range 0.05–0.25 mg/mL as described by Jamdar and colleagues [33]. Each antioxidant activity assay was conducted in triplicate at a specific protein concentration for all samples.

### 2.8. Data Analysis

All measurements were conducted in triplicate. Data were analyzed by ANOVA and Duncan Multiple Range Comparison at 5% significance level using SAS 9.4 (SAS Institute, Cary, NC, USA).

## 3. Results

### 3.1. Effect of Alcalase Treatment on the Allergen Contents Peanuts

**Figure 1:**
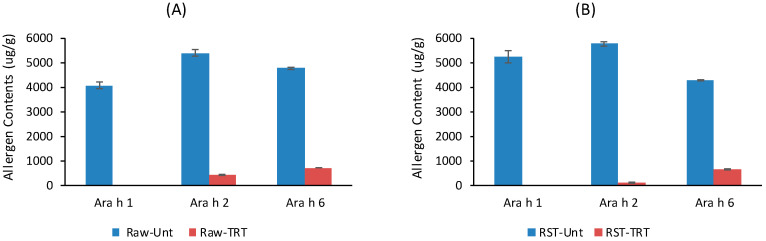
The concentrations of major allergen residues in peanut protein extracts ((**A**) raw peanuts, (**B**) dry roasted peanuts, RST—roasted, Unt—untreated, TRT—treated, Alcalase/Peanut = 3.5% (*v*/*w*), treatment time = 3 h).

Figure 1 shows that treatment of raw peanuts with 3.5% Alcalase (*v*/*w*) eliminated the majority of Ara h 1 in both raw and roasted peanuts, but small amount of Ara h 2 and significant amount of Ara h 6 remained. The quantities of residue Ara h 2 and Ara h 6 in raw peanuts were higher than those in the roasted peanuts. This suggests that Ara h 2 and Ara h 6 in raw peanuts are more resistant to Alcalase hydrolysis, and longer hydrolysis time or higher protease concentration may be needed to eliminate these two allergens. In addition, the ELISAs are designed to detect intact proteins from peanut (Ara h 1, 2, or 6). Therefore, these ELISAs may not detect smaller fragments of the allergens, which may be still allergenic.

### 3.2. Effect of Alcalase Treatment on the In Vitro Allergenicity of Raw Peanuts

**Figure 2:**
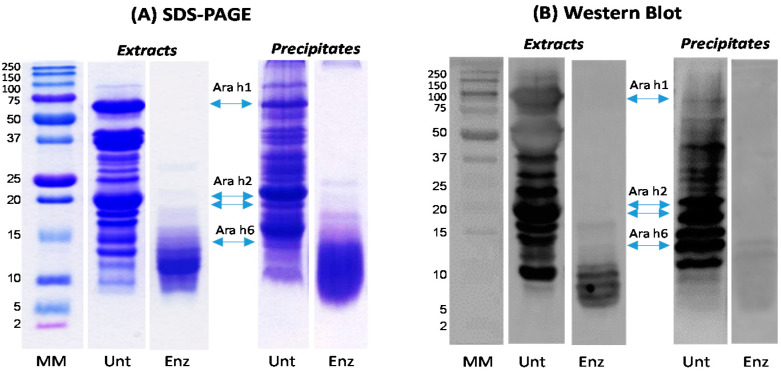
SDS-PAGE (**A**) and Western blot (**B**) of Alcalase treated raw peanut samples: MM—molecular marker, Unt—untreated, Enz—enzyme treated (Alcalase/Peanut: 3.5% (*v*/*w*), treatment time: 3 h, protein loaded: 25 µg/well).

The SDS-PAGE and Western blot of untreated sample and sample treated with 3.5% Alcalase further confirmed the data shown in Figure 1. The protease treatment eliminated the protein bands of Ara h 1 and greatly reduced Ara h 2 and Ara h 6, although they were still visible (Figure 2A). The residues Ara h 2 and Ara h 6 still had ability to bind to the IgE antibody in the pooled plasma but the intensity of binding was obviously weaker compared with those in the untreated peanuts (Figure 2B). However, Figure 2 also shows that the hydrolysis of original allergens generated some fragments/peptides with molecular weight 5–10 kDa (Figure 1A), and these peptides showed strong IgE-binding, particularly those in the soluble portion of the peanuts (Figure 2B). The results indicate that treatment of raw peanuts with 3.5% Alcalase greatly reduced the allergenic potential of peanuts, but some peptides formed during protein hydrolysis are immunoreactive.

### 3.3. Effects of Protease Treatment on the Peroxide Value of Peanuts during Storage

**Figure 3:**
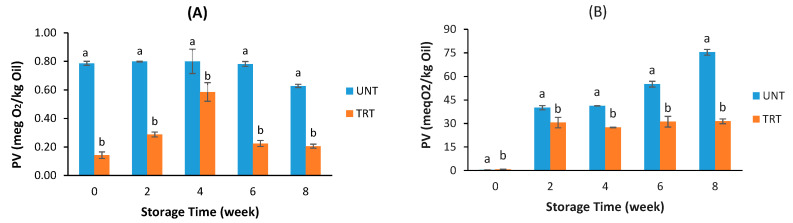
Effects of protease treatment on peroxide values of raw (**A**) and roasted (**B**) peanuts during storage (UNT—untreated, TRT—enzyme treated). At same storage time, the data bars with different letters are significantly different at *p* < 0.05.

The PV of Alcalase treated raw peanuts (Raw-TRT) was lower than that of untreated raw peanuts (Raw-Unt) during the 8-week storage period; it increased with storage time and reached the highest at week 4, and then decreased with further increase of storage time (Figure 3A). The PV of Raw-Unt was obviously higher than that of Raw-TRT over the whole storage period; it remained unchanged from week 0 to week 6 and significantly decreased at week 8. Dry-roasted peanuts showed much higher PV than the raw at same storage time (Figure 3B). The PV of untreated (RST-Unt) and Alcalase treated dry-roasted peanuts (RST-TRT) were 0.4 and 0.67 at week 0, but these values increased to 40.07 and 30.53, respectively, by week 2. The PV of RST-TRT then remained constant through the storage, while the PV of RST-UNT increased quickly with storage time and reached 75.4 by week 8. The PV is a quantitative measurement of hydroperoxides formed in the induction period of lipid oxidation, but the hydroperoxides are unstable, they decompose to form different aldehydes. The decrease of PV after reaching the peak value indicates the degradation rate of hydroperoxide exceeded the formation rate.

### 3.4. Effects of Protease Treatment on the TBARS of Peanuts during Storage

**Figure 4:**
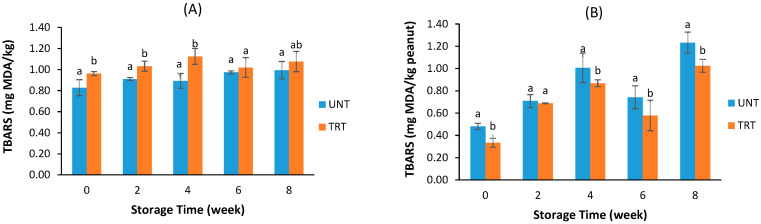
Effects of protease treatment on thiobarbituric acid reactive substances (TBARS) of raw (**A**) and roasted (**B**) peanuts during storage (UNT—untreated, TRT—enzyme treated). At same storage time, the data bars with different letters are significantly different at *p* < 0.05.

The initial TBARS of Alcalase-treated raw peanuts was higher than that of untreated (*p <* 0.05), reaching the highest at week 4 then went down, while the TBARS of raw untreated peanuts steadily increased with storage time and became the same as that of treated peanuts at weeks 6 and 8 (Figure 4A). The pattern of TBARS change in Alcalase treated raw peanuts corresponded to the increase of PV as shown in Figure 3A. The higher initial TBARS of the Alcalase treated raw peanut samples might be due to post-enzyme treatment drying because the treated peanuts were vacuum dried for 18 h at 75 °C to remove the moisture. The TBARS of dry-roasted peanuts treated with Alcalase were significantly lower than that of untreated at same storage time (*p <* 0.05), and the changes of TBARS were in the same trend for both untreated and treated samples (Figure 4B). Old peanut smell was detected at weeks 5 and 6 for untreated raw and roasted peanuts, at week 7 for treated roasted peanuts, but not detected in treated raw peanuts. Therefore, protease treatment not only reduced allergen content, but also slowed down oxidation, thus may extend the shelf life of peanuts.

### 3.5. Antioxidant Activity of Peanut Extracts

**Figure 5:**
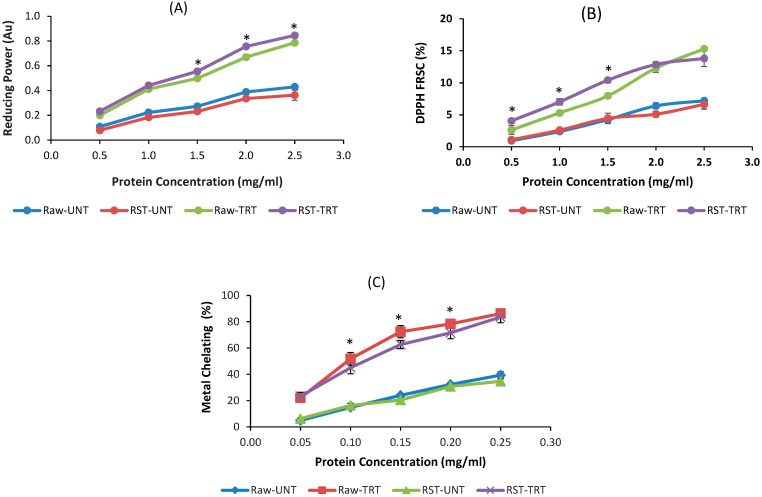
Effects of protease treatment on antioxidant activity of peanuts. (**A**) Reducing power, (**B**) DPPH free radical scavenging capacity, (**C**) metal chelating capacity (UNT—untreated, TRT—treated, Raw—raw peanuts, RST—roasted peanuts). The points with asterisks indicate significantly different values between Raw–TRT and RST–TRT.

Figure 5 shows that the Alcalase treatment significantly increased the antioxidant activities of peanuts. The reducing power, DPPH free radical scavenging capacity, and metal chelating increased almost linearly with protein concentration; the difference in these antioxidant properties between Alcalase treated and untreated peanuts also increased with protein concentration. The reducing power of raw untreated peanut extract was slightly higher than that of roasted untreated peanut extract at same protein concentration, but it was vice versa for the Alcalase treated peanuts (*p* < 0.05) (Figure 5A). There were no significant differences in the DPPH free radical scavenging and metal chelating capacities between untreated raw and roasted peanut extracts; the Alcalase treated RST showed higher DPPH free radical scavenging than Alcalase treated Raw at same protein concentration, while the metal chelating capacity was vice versa (Figure 5B,C). The increased antioxidant activity may explain the lower PV of Alcalase treated raw and roasted peanuts shown in Figure 3.

## 4. Discussions

Oxidative stability and allergenicity are two major concerns of peanuts. While protease hydrolysis of peanuts has shown to be effective and practical in reducing allergen content and allergenicity of roasted and raw peanuts [29,30,31,32], the effect of hydrolysis on the oxidative stability has not been investigated. Oxidation deterioration limits the shelf life, reduces the nutritional value, and renders peanuts unacceptable by consumers because of the rancid flavor. In this study, raw and roasted peanut kernels were treated with Alcalase, the most effective protease, under best enzymatic treatment conditions established in the previous study [30]. The changes of two oxidation indicators, PV and TBARS, during storage and the change of antioxidant activity of peanut protein extract due to protease treatment were determined.

The results further confirm that Alcalase hydrolysis of peanut kernels greatly reduced the overall IgE-binding of both soluble and insoluble portions of peanuts although some protein residues and fragments (5–15 kDa) retained the ability to bind IgE in the plasma of peanut allergic patients (Figure 2). The residue immunoreactive proteins/fragments could include small quantity of Ara h 6 (14.5 kDa) as shown in Figure 1, lipid transfer proteins Ara h 9 (9.8 kDa), Ara h 16 (8.5 kDa), Ara h 17 (11 kDa), and defensin proteins Ara h 12 (5.2 kDa) and Ara h 13 (5.5 kDa) [38,39]. The results suggest the presence of epitopes in the fragments and more extensive hydrolysis may be needed to remove those epitopes.

The oxidative stability test was conducted at 37 °C to accelerate the oxidation, so that the difference in oxidation status could be detected in a relatively short period [40], and to mimic storage conditions of hot areas such as Southeast Asia, West and Central Africa, where air conditioning is not available and peanuts are usually stored at ambient temperature. The results of this study show that the PV of Alcalase treated peanuts were obviously lower than that of untreated ones for both raw and roasted through the storage period of this study (Figure 3). The PVs of untreated roasted peanuts at the beginning, week 4, and week 8 of storage are comparable to those reported by a group of Japanese scientists [17]. It is well known that PV is the measurements of hydroperoxides formed at the initial stage of autoxidation [13], but the hydroperoxides are unstable compounds, and they further decompose to form different aldehydes which contribute to the off-flavor/rancidity of peanuts [21].

Although the PV of protease treated peanuts was lower than that of untreated at the same storage time, the TBARS of Alcalase treated raw peanuts were slightly but significantly higher than that of untreated, particularly, in the early storage stage (Figure 4A). This might be caused by the 18 h drying at 72 °C in the dehydrator. It was reported that the oxidation of peanut oil linearly increased and the concentration of polyunsaturated fatty acid decreased with heating time [41]. However, at the later stage of storage, the difference in TBARS between Alcalase treated and untreated samples became smaller, which indicates the faster increase of TBARS in untreated samples. For roasted peanuts, the TBARS of treated samples were lower than those of untreated over the storage period although the patterns of TBARS change with storage time was the same (Figure 4B). This suggests that the Alcalase treatment of roasted peanuts did not accelerate TBARS formation during storage.

The present study also show that protease treatment of peanut kernels increased the reducing power, DPPH free radical scavenging activity, and metal chelating activity of peanut protein extracts (Figure 5). Similar results were reported for hydrolyzed hemp protein [42] and peanut protein hydrolysate [33] with exception of the reducing power of peanut protein hydrolysate in study of Jamdar and colleagues. The increase of antioxidant activity of peanut extract by Alcalase treatment is due to the formation of antioxidant peptides. Protease hydrolysis is one of the most important methods to produce antioxidant peptides and other bioactive peptides from food protein [43,44]. The antioxidant potential of food-derived peptides is greatly dependent on the amino acid sequence and the length of peptides with many identified antioxidant peptides containing up to 10 amino acids, some up to 15 [45]. The molecular weights of peanut proteins are in the range of 10–70 kDa (Figure 2), and extensive hydrolysis is needed to produce peptides smaller than 10 amino acids. The increased antioxidant activity of peanut protein extracts may explain the lower PV and relative stable TBARS of Alcalase treated peanuts.

## 5. Conclusions

Oxidative stability and allergenicity are two major issues of peanuts due to the high content of unsaturated lipid and the presence of potent allergenic proteins. The present study further confirms that Alcalase hydrolysis of peanut kernels greatly reduced the overall allergenic potential of raw and roasted peanuts although some fragments formed during protein hydrolysis retained significant immunoreactivity. However, Alcalase treatment reduced the PV and did not accelerate TBARS formation in both raw and roasted peanuts during storage at 37 °C. Thus, it is safe to say that Alcalase treatment did not significantly affect the oxidative stability, thus the shelf-life of peanut kernels. This is most likely attributed to the formation of antioxidant peptides through enzymatic degradation of protein during protease treatment because the extracts of Alcalase treated peanuts showed much higher antioxidant activity than those of untreated peanuts. Research on the impacts of protease treatment on the nutrient composition and sensory quality of peanuts is needed.
